# Effects of salinomycin and ethanamizuril on the three microbial communities *in vivo* and *in vitro*

**DOI:** 10.3389/fmicb.2022.941259

**Published:** 2022-08-12

**Authors:** Xiaolei Cheng, Haihong Zheng, Chunmei Wang, Xiaoyang Wang, Chenzhong Fei, Wen Zhou, Keyu Zhang

**Affiliations:** Key Laboratory of Veterinary Chemical Drugs and Pharmaceutics, Ministry of Agriculture and Rural Affairs, Shanghai Veterinary Research Institute, Chinese Academy of Agricultural Sciences, Shanghai, China

**Keywords:** microbial community, salinomycin, ethanamizuril, metabolite profile, physiological profiling

## Abstract

The fate of a drug is not only the process of drug metabolism *in vivo* and *in vitro* but also the homeostasis of drug-exposed microbial communities may be disturbed. Anticoccidial drugs are widely used to combat the detrimental effects of protozoan parasites in the poultry industry. Salinomycin and ethanamizuril belong to two different classes of anticoccidial drugs. The effect of salinomycin and ethanamizuril on the microbiota of cecal content, manure compost, and soil remains unknown. Our results showed that although both salinomycin and ethanamizuril treatments suppressed some opportunistic pathogens, they failed to repair the great changes in chicken cecal microbial compositions caused by coccidia infection. Subsequently, the metabolite5 profiling of cecal content by LC-MS/MS analyses confirmed the great impact of coccidia infection on chicken cecum and showed that histidine metabolism may be the main action pathway of salinomycin, and aminoacyl tRNA biosynthesis may be the major regulatory mechanism of ethanamizuril. The microbial community of manure compost showed a mild response to ethanamizuril treatment, but ethanamizuril in soil could promote *Actinobacteria* reproduction, which may inhibit other taxonomic bacteria. When the soil and manure were exposed to salinomycin, the *Proteobacteria* abundance of microbial communities showed a significant increase, which suggested that salinomycin may improve the ability of the microbiota to utilize carbon sources. This hypothesis was confirmed by a BIOLOG ECO microplate analysis. In the animal model of coccidia infection, the treatment of salinomycin and ethanamizuril may reconstruct a new equilibrium of the intestinal microbiota. In an *in vitro* environment, the effect of ethanamizuril on composting and soil microbiota seems to be slight. However, salinomycin has a great impact on the microbial communities of manure composting and soil. In particular, the promoting effect of salinomycin on *Proteobacteria* phylum should be further concerned. In general, salinomycin and ethanamizuril have diverse effects on various microbial communities.

## Introduction

The microbiome is a unique ecosystem. Surfaces of animals, plants, and soil are colonized by complex microbial communities whose structure and function are seriously affected by microbe–microbe, microbe–host, and microbe–environmental factors interactions. Growing attention has been paid to the factors that influence the assembly and stability of host-associated microbiomes and their impact on host phenotype, ecology, and evolution over the last decades (McDonald et al., 2020). The animal gut microbiome is a dynamic collection of bacteria, archaea, fungi, and phages that plays numerous roles in immune development, pathogen colonization resistance, and food metabolism. Although the ecology of the gut microbiome is relatively stable, it could be impacted by various factors, such as food, pathogens, and drugs (Schwartz et al., 2020). The soil microbiome is clearly a key component of natural ecosystems. A large amount of evidence highlights that the survival and growth of soil microorganisms are seriously limited by abiotic stressors (e.g., acidic conditions) and frequent interference of other soil microbial groups (antibiotic-producing bacteria) (Islam et al., 2020). Composting is an environmental-friendly approach to transform animal manure into high-quality agricultural organic fertilizer (Wan et al., 2021). During composting, microbiota are an important driver of organic matter depolymerization and are influenced by physicochemical and other factors.

Coccidiosis is a serious protozoal infectious disease and is estimated to cause global economic losses of up to $3 billion annually only in the poultry industry (Kadykalo et al., 2018; Zhang et al., 2020a). The main method of controlling coccidiosis relies heavily on the use of the highly effective coccidiostats reasonably and correctly. Predominant drugs in controlling coccidiosis are polyether ionophore antibiotics and triazines compounds. Polyether ionophore antibiotics have the activity of facilitating the transport of cations through the cell membranes of target organisms (including protozoa and Gram-positive bacteria) and are important coccidiostats (Antoszczak et al., 2019). Salinomycin, a monocarboxylic polyether antibiotic, is an important member of the ionophore anticoccidials in poultry production. As broad-spectrum ionophore anticoccidials with activity against Gram-positive bacteria, fungi, and coccidia, salinomycin is widely used in veterinary medicine worldwide (Zhou et al., 2013). In addition, triazine compounds have been safely and effectively used to combat the detrimental effects of protozoan parasites, especially poultry coccidiosis, in the global veterinary community for years. Triazines have been reported to inhibit the nuclear division of protozoan parasites, thereby interfering with the development and growth of schizonts and gametocytes (Stock et al., 2018). Ethanamizuril is a novel triazine compound that has shown excellent effectiveness against coccidiosis at a dosage of 10 mg/L in drinking water (Wang et al., 2020; Zhang et al., 2020b). It has been approved for usage in broiler chickens since 2020 in China.

The resistance and resilience of a microbiome to external stress and perturbation are among the potential properties that characterize a healthy microbiome (Lloyd-Price et al., 2016). Accumulating evidence suggests that antibiotics play complex roles by reshaping the intestinal microbiota. Deep sequencing of the bacterial 16S rRNA gene revealed that the richness of the cecal microbiota was significantly reduced while the evenness increased after the male Cobb broilers had been supplemented with subtherapeutic salinomycin as antimicrobial growth promoters for 14 days (Robinson et al., 2019). The interaction between microbiota and used agents is complex and bidirectional (Weersma et al., 2020). The metabolic fates of drugs in the intestinal tract, manure compost, and soil environment have always been widely concerned. Understanding the microbial community profile is also important for predicting the fate of drugs in the environment. However, so far, the effect of coccidiostats on the gut microbiota of coccidial-infected chickens remains unknown. Moreover, excretion of the prototype drug through feces is the main metabolic pathway for ethanamizuril and salinomycin *in vivo* after chickens are administered (Henri et al., 2012; Liu et al., 2020). To the best of our knowledge, there is no research data on the effects of salinomycin and ethanamizuril on manure composting and soil microbial communities.

In the present study, we investigated and compared the effects of salinomycin and ethanamizuril on the microbiota of coccidia-infected broilers' cecal content, manure composting, and soil using the bacterial 16S rRNA gene sequencing. The two coccidiostats were chosen because they belong to different classes of anticoccidial drugs and are known to have different antiprotozoan mechanisms. Furthermore, the effects of salinomycin and ethanamizuril on the metabolism of the chicken cecum and on soil microbiota function were revealed by LC-tandem mass spectrometry and BIOLOG ECO microplates, respectively. Identification of a number of bacterial compositions that are altered in response to salinomycin and ethanamizuril sheds new light on their anticoccidial mechanism *in vivo* and harmless elimination *in vitro* and may contribute to the use of microbiota to improve anticoccidial efficiency and environmental protection in the future.

## Materials and methods

### Drugs and parasites

Salinomycin sodium was purchased from China Institute of Veterinary Drug Control (Beijing, China). Ethanamizuril and *E. tenella* strain (CAAS2111606) was obtained from Shanghai Veterinary Research Institute, Chinese Academy of Agriculture Sciences (Shanghai, China). The oocysts of *E. tenella* were sporulated and purified according to the methods described in our previous studies (Li et al., 2019). Analytical grade chemicals and solvents for extraction and analysis were purchased from local chemical suppliers.

### Experimental design

#### Exposure to the cecum *in vivo*

All the research was approved by the Shanghai Veterinary Research Institutional Animal Care Committee and in accordance with the National Institutes of Health Guide on the Care and Use of Laboratory Animals. Then, 1-day-old healthy Pudong yellow broiler chickens were obtained from the same hatch of a local commercial hatchery and reared in wire cages sterilized by dry heat sterilization. Feed and water were supplied *ad libitum*, and no additive drugs were used. At 8 days of age, a total of 120 chickens were weighed and randomly divided into four groups of 10 birds per cage. Each group was separated to prevent accidental cross-infection. The four groups were the control group (Mock), the non-medicated control group (NC), the ethanamizuril group (L1), and the salinomycin group (S). Then, the ethanamizuril group (L1) was treated with 10 mg/L of ethanamizuril in drinking water for 3 days, and the salinomycin group (S) was treated with 60 mg/kg salinomycin in feed for 7 days. Except for the chickens in the control group, all chickens were inoculated with 8 × 10^4^ *E. tenella* sporulated oocysts per chicken at 10 days of age. The chickens in every group were all sacrificed 7 days post-infection. The cecal contents of each individual were aseptically collected and immediately immersed in liquid nitrogen. Ten samples randomly selected from each group were used for microbiota diversity analysis and six for metabolomic analysis.

#### Exposure on manure composting

At 12 days of age, chickens were randomly assigned to three groups: the control group (BLANK), the ethanamizuril group (EZL), and the salinomycin group (SAL). Each group was reared in wire cages and separated to prevent accidental cross-infection. The control group was not given any antibiotics or anticoccidial drugs. The ethanamizuril group was treated with 10 mg/L of ethanamizuril in drinking water, and the salinomycin group was treated with 60 mg/kg of salinomycin in feed every day. Fresh fecal samples from 10 individuals in each group were collected at 1–3 days and were mixed well. About 80.0 g of fecal sample was weighed into a 250-ml sterile bottle, and the bottle was sealed with a breathable film and placed in an incubator. To simulate manure composting in the laboratory, the temperature of the incubator was set to be constant at 45°C after ramping up (30°C on day 1, 40°C on day 2, 50°C on days 3–7, and 45°C on days 7–11). The samples of manure composting on days 0, 7, and 11 were aseptically collected and immediately immersed into liquid nitrogen for microbiota diversity analysis. Meanwhile, the water content, pH, and electrical conductivity of samples were detected. Each treatment was replicated three times.

#### Exposure on soil

Loam soil (pH 8.1, electrical conductivity 70.52 μS/cm) was obtained from a farmland (Huai'an, China). After passing through a 2-mm sieve, the fresh loam was divided into the three groups: the control group, the ethanamizuril group, and the salinomycin group. The control group was not given any antibiotics or anticoccidial drugs. The loam of the ethanamizuril group and the salinomycin group was fully mixed with ethanamizuril and salinomycin. The final concentrations of ethanamizuril and salinomycin in the mixture were 2.5 and 0.1 mg/kg, respectively. Each 2 kg of loam was placed in a flowerpot and placed in the same natural environment for 20 days. The samples of loam were aseptically collected with the five-point sampling method and immediately immersed in liquid nitrogen for a microbiota diversity analysis. The samples included the day 0 control group (BLANK 0day), the day 20 control group (BLANK 20day), the day 20 ethanamizuril group (ZEL 20day), and the day 20 salinomycin group (SAL 20day). Each treatment was replicated three times.

### Microbiome profiling by 16S rRNA

To evaluate microbial profile, total genome DNA from samples was extracted using the TIANamp Soil DNA Kit (TIANGEN BIOTECH, China). Agarose gel electrophoresis was used to monitor DNA concentration and purity. Qualified DNA was diluted to 1 μg/μl using sterile water. A pair of primers (338F: 5'-ACTCCTRCGGGAGGCAGCAG-3' and 806R: 5'-GGACTACCVGGGTATCTAAT-3') was used to amplify the V3-V4 hypervariable region of the 16S rRNA gene (Huang et al., 2018). According to the manufacturer's recommendations, the TruSeq^®^ DNA PCR-Free Sample Preparation Kit (Illumina, USA) was used to generate sequencing libraries. The quality of the sequencing libraries was assessed and sequenced on an Illumina NovaSeq platform.

A sequence analysis was conducted as previously described (Zhou et al., 2020). Briefly, FLASH was used for assembling the paired-end raw reads obtained from the 16S ribosomal amplicon sequencing and barcode identification for de-multiplexing. To obtain the high-quality clean tags, the raw tags were filtered according to the QIIME quality-controlled process. Next, chimeric sequences were identified and removed using the UCHIME algorithm, and the finally obtained effective tags were clustered into operational taxonomic units (OTUs). According to the sequence analysis with UPARSE software, the sequences with a similarity greater than a threshold (97%) were assigned to the same OTUs. Furthermore, based on the Mothur algorithm, each representative sequence of each OTU was annotated using the Silva database. Multiple sequence alignments were conducted using the MUSCLE software. To assess the impacts of two anticoccidials on the microbiota, both alpha diversity analysis and beta diversity analysis were performed based on the normalized OTUs abundance information. All indices in alpha diversity analyses including Observed-species, Chao1, Shannon and Simpson indices, ACE, and Good's coverage were obtained using QIIME software (version 1.9.1) and displayed with R software (version 2.15.3). Beta diversity on both weighted and unweighted UniFrac was analyzed in QIIME software (version 1.9.1).

### Cecal metabolomic profiling

We used the mixed solvent extraction method to obtain metabolites from the cecal contents. Briefly, a 25-mg sample was mixed with 500 μl of fresh-made LC/MS-grade extract solution in an EP tube (methanol/ acetonitrile/ water = 2: 2: 1, with the isotopically-labeled internal standard mixture). The mixture was homogenized at 35 Hz for 4 min and sonicated for 5 min in an ice-water bath 3 times repeatedly. Then, the mixture was incubated for 1 h at −40°C and centrifuged at 12,000 rpm for 15 min at 4°C. Afterward, each supernatant was moved to a fresh EP tube for the LC–MS/MS analysis. The quality control (QC) sample was prepared by mixing an equal aliquot of the supernatants from all samples.

To detect the metabolites of cecal contents, 3 μl of the extracted supernatant was injected into an LC-MS/MS system which consisted of a UHPLC system (Vanquish, Thermo Fisher Scientific) with a UPLC BEH Amide column (2.1 × 100 mm, 1.7 μm) coupled to Q-Exactive HFX mass spectrometer (Orbitrap MS, Thermo Fisher Scientific). The mobile phase of the system was a binary solvent mode consisting of 25 mmol/L ammonium acetate and 25 mmol/L ammonia hydroxide in water (pH = 9.75) (A) and acetonitrile (B). The auto-sampler temperature was set at 4°C.

According to the ability to acquire MS/MS spectra on information-dependent acquisition (IDA) mode in the control of the acquisition software (Xcalibur, Thermo), mass spectrometry of metabolites was performed using a Q-Exactive HFX mass spectrometer. In IDA mode, the acquisition software continuously evaluated the full scan MS spectrum. Furthermore, the ESI conditions were set as follows: sheath gas flow rate as 30 Arb, Aux gas flow rate as 25 Arb, full MS resolution as 6.0 × 10^4^, MS/MS resolution as 7.5 × 10^3^, capillary temperature 350°C, collision energy as 10/30/60 in NCE mode, and spray voltage as 3.6 kV (positive) or −3.2 kV (negative), respectively.

After the LC–MS/MS analysis, the raw mass spectrometry data were converted to the mzXML format by ProteoWizard software and processed with an in-house program (BiotreeDB), which was developed using R and based on XCMS for peak detection, extraction, alignment, and integration. The information of normalized total peak intensity was then exported to SIMCA (version 16.0.2) software package where principal component analysis (PCA) and orthogonal partial least squares discriminant analysis (OPLS-DA) of the multivariate data were performed. The threshold for visualizing the distribution and the grouping of the samples was located at 97% in PCA. The importance of each variable in the OPLS-DA model in the projection (VIP) value was further calculated to show its contribution to the classification. Metabolites meeting VIP > 1 and *p* ≤ 0.05 were considered to be significantly differentially expressed metabolites (DEMs). Kyoto Encyclopedia of Genes and Genomes (KEGG) database (http://www.kegg.jp/) was used to further analyze the pathways of the metabolites.

### Analysis on physiological profiling of soil microbial community

To investigate the metabolic functional diversity of loam microbial communities which exposed salinomycin and ethanamizuril, the BIOLOG ECO microplate method was applied in this study. The fresh loam of the same origin was divided into three groups: the control group (BLANK), the ethanamizuril group (EZL), and the salinomycin group (SAL). The final concentrations of ethanamizuril and salinomycin in loam reached 10 and 60 mg/kg, respectively. A total of 300 g of loam sample was weighed into a 500-ml sterile bottle, and the bottle was sealed with a breathable film and placed in a dark incubator at 25°C. In total 12.5 g sample was evenly drawn from each bottle and placed in a new 250-ml sterile glass on 7, 14, 21, 28, and 35 days and then added into 90 ml of stroke-physiological saline solution. The mixture was oscillated and shaken at 200 rpm for 30 min at 4°C and then stewed for 15 min. In total 5 ml of the supernatant was transferred to 45 ml of sterile saline. This process was repeated two times to dilute the soil suspension to a 10^−3^ gradient. To each well of the BIOLOG ECO microplates (BIOLOG, United States), 150 μl of diluent was added, and the microplates were cultured at a constant temperature (25°C) for 10 days. During cultivation, the absorbance values of ECO microplates were read at 590 nm wavelength every 24 h using an enzyme label tester (Thermo Fisher, USA). Each treatment was replicated three times. Average well-color development (AWCD) on microplates indicated the metabolic activity of microorganisms in loam (Ge et al., 2018).

### Statistical analysis

All experimental data were presented as mean ± standard deviation. All data were statistically analyzed by Student's *t*-test. A *p* < 0.05 indicated significant differences.

## Results

### Impact on the cecal microbiota of infected chickens

**Figure 1:**
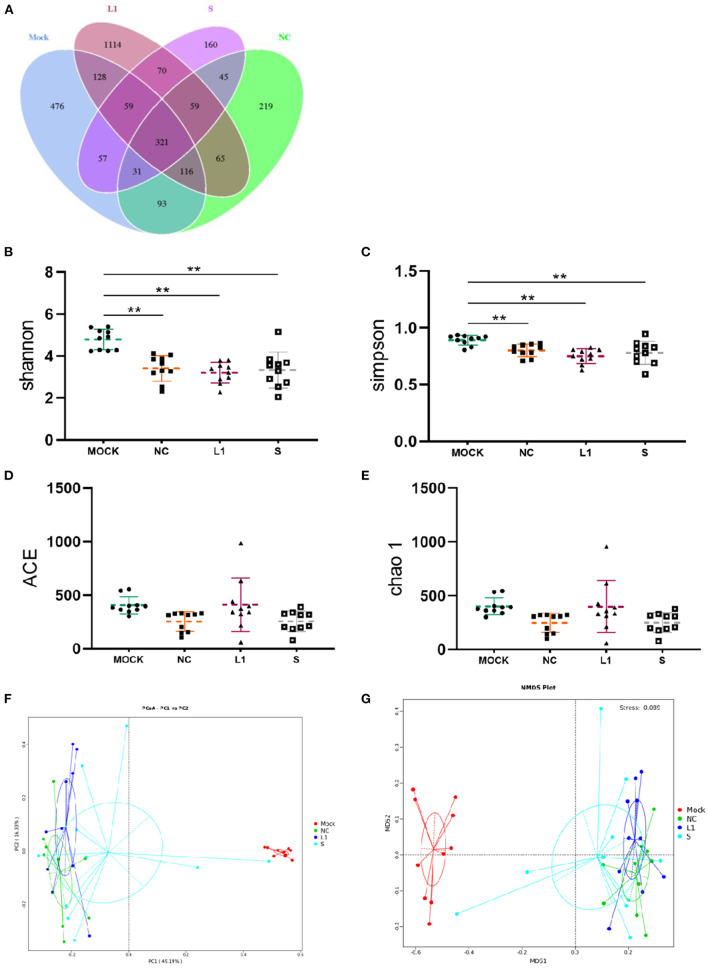
Venn diagram of shared OTUs of the different groups, as well as alpha diversity analysis and beta diversity analysis for the cecal microbiota of infected chickens. **(A)** for Venn diagram; **(B–E)** were the indices of Shannon, Simpson, ACE and Chao1, respectively. **(F)** for PCoA, **(G)** for NMDS. Mock for control group; NC for non-medicated control group; L1 for ethanamizuril group; S for salinomycin group. ^**^Shows the significantly different (*p* < 0.01) when the treated groups compared with control group.

The effect of salinomycin and ethanamizuril on the microbiota profiles of chicken cecum contents was analyzed by 16S ribosomal amplicon sequencing. In total, 3,013 OTUs were observed in the four experimental groups, of which 321 OTUs were common to every group (Figure 1A). Both the numbers of total and unique OTUs in the ethanamizuril group (L1) were the most, while those in the salinomycin group (S) were the least. Based on the OTUs, all alpha diversity indicators were calculated to evaluate the microbial richness and species biodiversity. All of Good's coverage index was >99%. As presented in Figures 1B–E, the Shannon and Simpson indices of the control group (Mock) were significantly higher than those of the other three groups (*p* < 0.05). There was no significant difference observed in the Chao1 and ACE indices. The beta diversity indicators [principal coordinates analysis (PCoA), non-metric multidimensional scaling (NMDS)] were computed to reflect the intragroup and intergroup distances. As shown in Figures 1F,G, the PCoA and NMDS plot revealed that there was remarkable discrimination in the cecal microorganism between the control group and the other three groups. The other three groups seemed to be closer to forming a loose cluster, but the points of the salinomycin group were still scattered.

**Figure 2:**
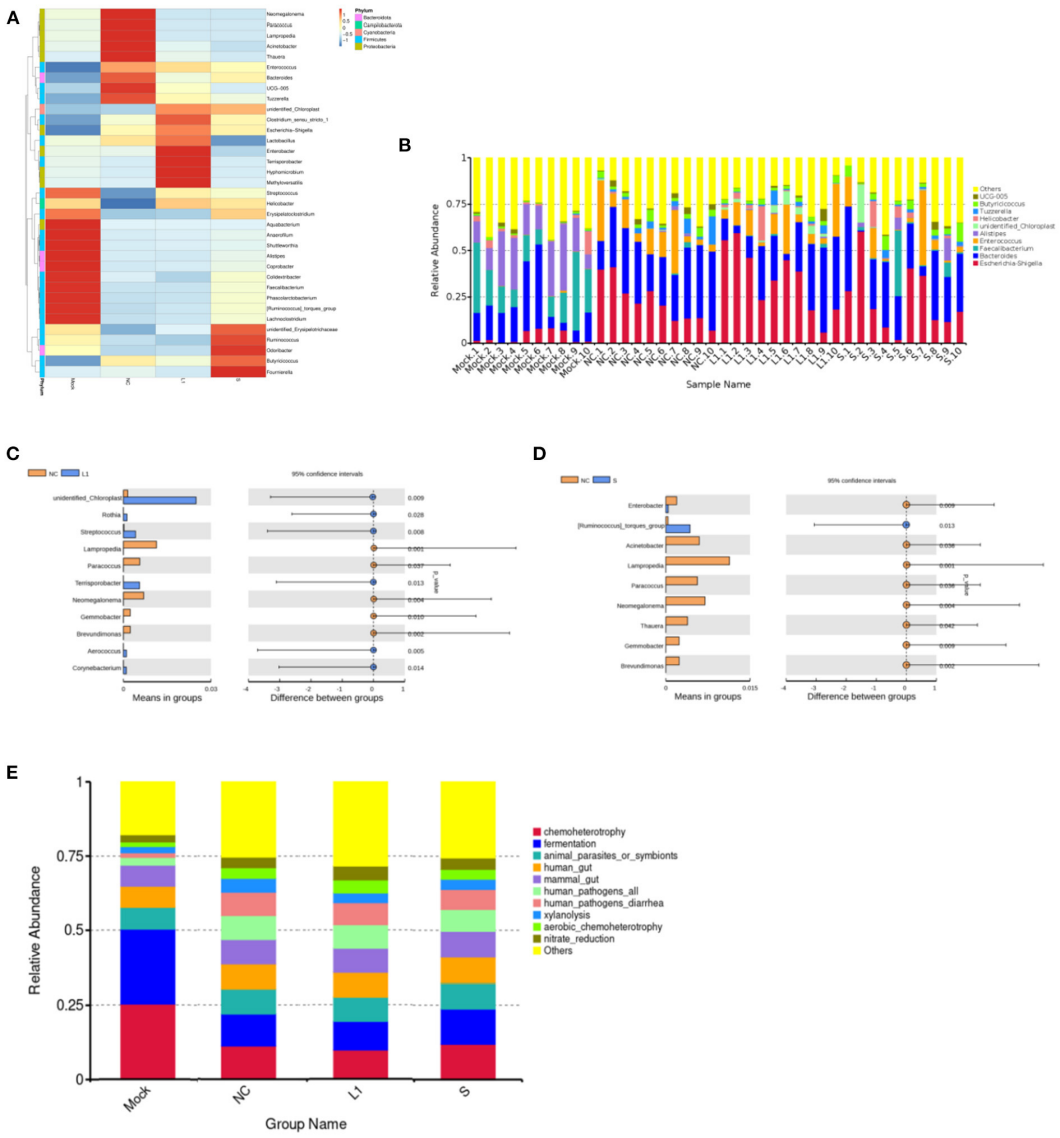
Analysis of the differences species and functions based on the OTUs abundant compositions for the cecal microbiota of infected chickens. **(A)** was the heat map of cluster analysis based on the level of genus. **(B)** was the composition of the top 10 microorganisms in the cecum at genus level. **(C)** was the analysis of significant differences species between ethanamizuril group vs. the non-medicated control group. **(D)** was the analysis of significant differences species between salinomycin group vs. the non-medicated control group. **(E)** was the analysis of microbial functions based on KEGG pathway of groups. Mock for control group; NC for non-medicated control group; L1 for ethanamizuril group; S for salinomycin group.

The heat map of the top 35 most abundant compositions in the microbiome community combined with their cluster analysis showed that there were wide variations in the bacterial taxa among groups (Figure 2A). The top 35 genera in relative abundance belong to five phyla, namely, *Firmicutes, Bacteroidota, Campilobacterota, Cyanobacteria, and Proteobacteria*. The relative abundance of genera was also compared predominantly (Figure 2B). Compared with the control group (Mock), the abundances of *Proteobacteria* (phylum level) and *Escherichia-Shigella* (genus level) increased significantly in the other three groups (*p* < 0.05), and the abundances of *Faecalibacterium* of *Firmicutes* and *Bacteroidota* of *Alistipes* decreased significantly (*p* < 0.05). In addition, it was worth noting that the abundance of some bacteria of *Firmicutes* such as *Tyzzerella* and *Enterococcus, Proteobacteria* such as *Acinetobacter*, and *Lampropedia* tended to be consistent with that of the healthy group after coccidiostats treatment. There was no significant difference in bacterial abundance between the salinomycin group and the ethanamizuril group. Furthermore, to distinguish the contribution of bacterial taxa to the difference in bacterial clustering, a *t*-test was performed to examine whether there was a significant difference between the non-medicated control group (NC) and the two coccidiostats treatment groups. As presented in Figures 2C,D, compared to the non-medicated control group (NC), the abundances of *unidentified Chloroplast, Rothia, Streptococcus, Terrisporobacter, Aerococcus*, and *Corynebacterium* were significantly increased in the ethanamizuril group, whereas *Lampropedia, Paracoccus, Neomegalonema, Gemmobacter*, and *Brevundimonas* decreased; *[Ruminococcus] torques group* in the salinomycin group were significantly increased and *Enterobacter, Acinetobacter, Lampropedia, Paracoccus, Neomegalonema, Thauera, Gemmobacter*, and *Brevundimonas* decreased. The significant difference between these bacteria made the top ten contributions to the significant difference between the two coccidiostats treatment groups and the control group.

The microbial function prediction analysis was conducted to determine the differences in the functions of microbiota among groups. According to Figure 2E, the top 10 functions include chemoheterotrophy, fermentation, animal parasites or symbionts, human gut, mammal gut, human pathogens, human pathogens diarrhea, xylanolysis, aerobic chemoheterotrophy, and nitrate reduction. The chemoheterotrophy and fermentation played a major role in the microbial function of cecal contents in the control group, which were significantly higher than those of the other three groups.

### Impact on the cecal metabolites of infected chickens

**Figure 3:**
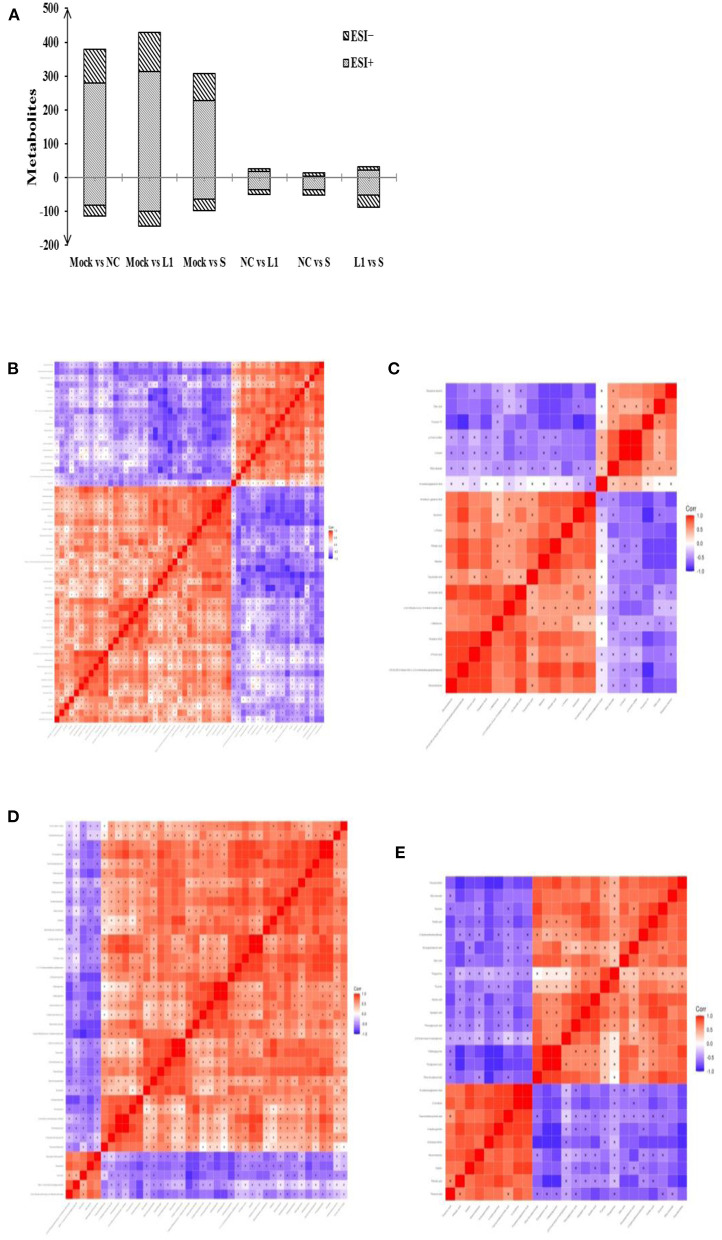
Total number of differentially expressed metabolites (DEM) in pairwise comparison and the heat map of correlation analysis for the cecal metabolites of infected chickens. **(A)** was the DEM distribution in pairwise comparison. **(B,C)** for the pairwise comparison of ethanamizuril group vs. the non-medicated control group in positive and negative mode, respectively; **(D,E)** for the pairwise comparison of salinomycin group vs. the non-medicated control group in positive and negative mode, respectively. Mock for control group; NC for non-medicated control group; L1 for ethanamizuril group; S for salinomycin group.

The effect of salinomycin and ethanamizuril on the metabolite profiles of chicken cecum contents was analyzed by an untargeted LC–MS-based metabolomics platform. Both PCA and OPLS-DA score models showed significant differences in the distribution of metabolites among each group. According to the VIP > 1 and *p* < 0.05, detailed information about the DEM has been distinguished. As presented in Figure 3A, there were dramatic metabolic changes among the control group (Mock) vs. the non-medicated control group (NC), which indicated that coccidia infection led to a sharp increase in the number of DEM. Similar dramatic metabolic changes were also related to the control group (Mock) vs. the drug treatment groups. There were relatively few DEMs among the non-medicated control group (NC) and the drug treatment groups.

Compared to the non-medicated control group, there were 49 DEMs increased and 26 decreased in the ethanamizuril group (Figures 3B,C). These metabolites included glycerophospholipids {PC(14:0/14:0), lysoPC[18:1(9Z)]}, fatty acyls (oleic acid and elaidic carnitine), amines (1-butylamine, diethanolamine), amino acids, peptides, analogs (L-methionine, N6-acetyl-l-lysine, tyrosyl-lysine), pyrazoles (demethylated antipyrine), eicosanoids (11-dehydro-thromboxane B2), quaternary ammonium salts (phosphorylcholine), sesquiterpenoids (xanthorrhizol), pyrrolidinylpyridines (nicotine), aldehydes (acetamidopropanal), and others (o-cresol). The most up- and downregulated metabolites were xanthine and saccharin in the ethanamizuril group, respectively. Compared to the non-medicated control group, there were 51 metabolites increased and 14 decreased in the salinomycin group (Figures 3D,E). These metabolites included peptidomimetics (carnosine), amines (histidine), guanidines (methylguanidine), fatty acyls (ethyl stearate), purines and purine derivatives (guanine, 3-methyladenine), and others. The most up- and downregulated metabolites were porphobilinogen and daidzin in the salinomycin group, respectively. In addition, compared with the changes induced by ethanamizuril, the number of glycerophospholipids induced by salinomycin decreased.

The pathway enrichment analysis was used to find the key pathway with the highest correlation of metabolite differences. Table 1 shows the pathways of two coccidiostats that affect the metabolism of cecal contents in infected chickens. There were 8 most perturbed metabolic pathways including purine metabolism, aminoacyl-tRNA biosynthesis, glyoxylate and dicarboxylate metabolism, pentose phosphate pathway, TCA cycle, cysteine and methionine metabolism, arginine and proline metabolism, and glycerophospholipid metabolism in the comparison between the ethanamizuril group and the non-medicated control group. A total of 10 metabolic pathways, such as histidine metabolism, purine metabolism, and vitamin B6 metabolism, were disturbed in the comparison of the salinomycin group vs. the non-medicated control group. The metabolic pathways revealed by metabolomic profiling may be related to nitrate reduction, chemoheterotrophy, and fermentation functions which were revealed in microbiome profiling.

**Table 1:** Pathway analysis of different metabolites in the salinomycin group vs. non-medicated control group and the ethanamizuril group vs. non-medicated control group for the cecal microbiota of infected chickens.

**Group**	**Pathway**	**Total**	**Hits**	**Raw** ***p***	**Impact**
Ethanamizuril group vs. non-medicated control group	Purine metabolism	63	3	0.026769	0.03958
	Aminoacyl-tRNA biosynthesis	44	2	0.042226	0
	Glyoxylate and dicarboxylate metabolism	19	1	0.13706	0.14286
	Pentose phosphate pathway	20	1	0.14378	0
	Citrate cycle (TCA cycle)	20	1	0.14378	0.05354
	Cysteine and methionine metabolism	27	1	0.18952	0.09849
	Arginine and proline metabolism	38	1	0.25697	0.07211
	Glycerophospholipid metabolism	33	1	0.30299	0.04037
Salinomycin group vs. non-medicated control group	Histidine metabolism	16	2	0.015352	0.0641
	Cyanoamino acid metabolism	6	1	0.071584	0
	Vitamin B6 metabolism	9	1	0.10555	0
	beta-Alanine metabolism	20	1	0.22037	0.07065
	Porphyrin and chlorophyll metabolism	27	1	0.28608	0
	Arginine and proline metabolism	38	1	0.37895	0.02677
	Pentose phosphate pathway	20	1	0.20808	0
	Biosynthesis of unsaturated fatty acids	22	1	0.22649	0
	Glutathione metabolism	26	1	0.26213	0.00835
	Purine metabolism	63	1	0.52645	0.00396

Total, the number of all metabolites in the pathway; Hits, the number of differential metabolites hitting the pathway; Raw p, p-value obtained by enrichment analysis; Impact, impact factor obtained from topology analysis.

### Impact on the fecal microbiota during manure composting

**Figure 4:**
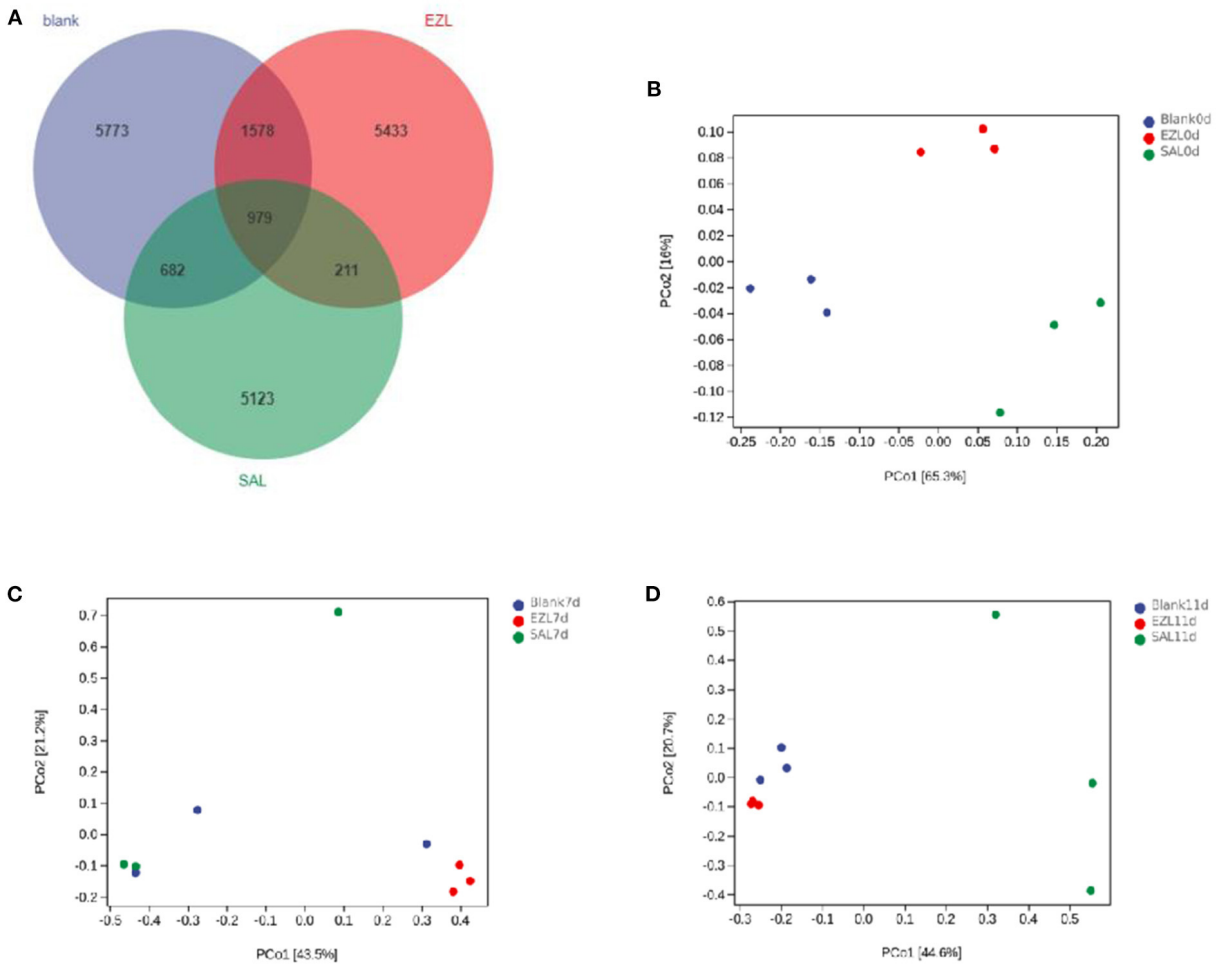
Venn diagram of shared OTUs of the different groups, and Principal coordinates analysis (PCoA) of days 0, 7 and 11 of the structure of the microbiota during manure composting. Blank was the control group; SAL was the salinomycin group; ZEL is the ethanamizuril group. **(A)** for Venn diagram; **(B–D)** were PCoA for the day 0, 7 and 11, respectively.

During manure composting, the values of pH and electrical conductivity of fecal samples were significantly increased, but the water content had no significant change (Supplementary Table 1). The 16S rRNA gene-based sequencing generated from fecal samples on days 0, 7, and 11 yielded a total of 2,108,973 high-quality reads. The average assembled 16S (V3–V4) length was 428 nucleotides, ranging from 215 bp to 433 bp. In total, 19,779 OTUs were observed in the three treatment groups, of which 979 OTUs were common to every group (Figure 4A). Both the numbers of total and unique OTUs in the control group (BLANK) were the most, while those in the salinomycin group (SAL) were the least.

As presented in Table 2 all of Good's coverage index was >99%. On day 0, the Shannon index of the control group (BLANK) was significantly higher than those of the ethanamizuril group (EZL) and salinomycin group (SAL) (*p* < 0.05), and Faith's PD index of the control group and salinomycin group (SAL) was significantly higher than those of th ethanamizuril group (EZL) (*p* < 0.05). There was no significant difference observed in other indices and other days. PCoA was used to evaluate sample grouping tendency on the same day in taxa (Figure 4). The PCoA score plot showed the clear separation of the control group, the ethanamizuril group, and salinomycin group on day 0 (Figure 4A). Similar separation of groups was also seen in the research of ethanamizuril and salinomycin on cecal microbiota (Figures 1F,G). On days 7 and 11, the plot of the ethanamizuril group was gradually close to the control group (Figure 4C), which meant great variation occurred during manure composting.

**Table 2:** Alpha diversity indexes of the fecal microbiota during manure composting.

**Time (day)**	**Group**	**Observed_species**	**Shannon**	**Simpson**	**Chao1**	**Faith_pd**	**Goods_coverage**
0	Blank	1,209.73 ± 109.70	4.68 ± 0.17^a^	0.77 ± 0.03	1,396.23 ± 139.10	59.53 ± 2.50^a^	0.9932 ± 0.0010
	EZL	744.67 ± 43.85	3.27 ± 0.21^b^	0.61 ± 0.04	877.55 ± 47.23	43.56 ± 1.45^b^	0.9956 ± 0.0002
	SAL	1,115.90 ± 52.21	3.84 ± 0.50^b^	0.64 ± 0.07	1,313.27 ± 41.51	56.16 ± 3.18^a^	0.9931 ± 0.0001
7	Blank	1,260.67 ± 1,377.22	5.72 ± 2.79	0.88 ± 0.14	1,338.92 ± 1,369.88	36.96 ± 25.83	0.9955 ± 0.0034
	EZL	1,955.47 ± 642.28	7.16 ± 1.28	0.93 ± 0.07	2,103.72 ± 616.12	59.51 ± 15.38	0.9909 ± 0.0014
	SAL	938.33 ± 845.39	4.80 ± 2.09	0.86 ± 0.09	1,078.57 ± 972.97	52.99 ± 59.15	0.9946 ± 0.0052
11	Blank	1,260.67 ± 1,377.22	7.45 ± 0.52	0.97 ± 0.01	2,216.01 ± 450.91	61.52 ± 13.18	0.9907 ± 0.0026
	EZL	1,955.47 ± 642.28	6.70 ± 0.42	0.94 ± 0.01	1,834.17 ± 392.71	61.54 ± 7.16	0.9910 ± 0.0026
	SAL	938.33 ± 845.39	5.80 ± 1.24	0.90 ± 0.09	1,389.14 ± 550.43	59.11 ± 39.38	0.9933 ± 0.0023

Blank, control group; EZL, ethanamizuril group; SAL, salinomycin group. Differentt letters in the same column showed statistically significant difference on the same day (p < 0.05).

**Figure 5:**
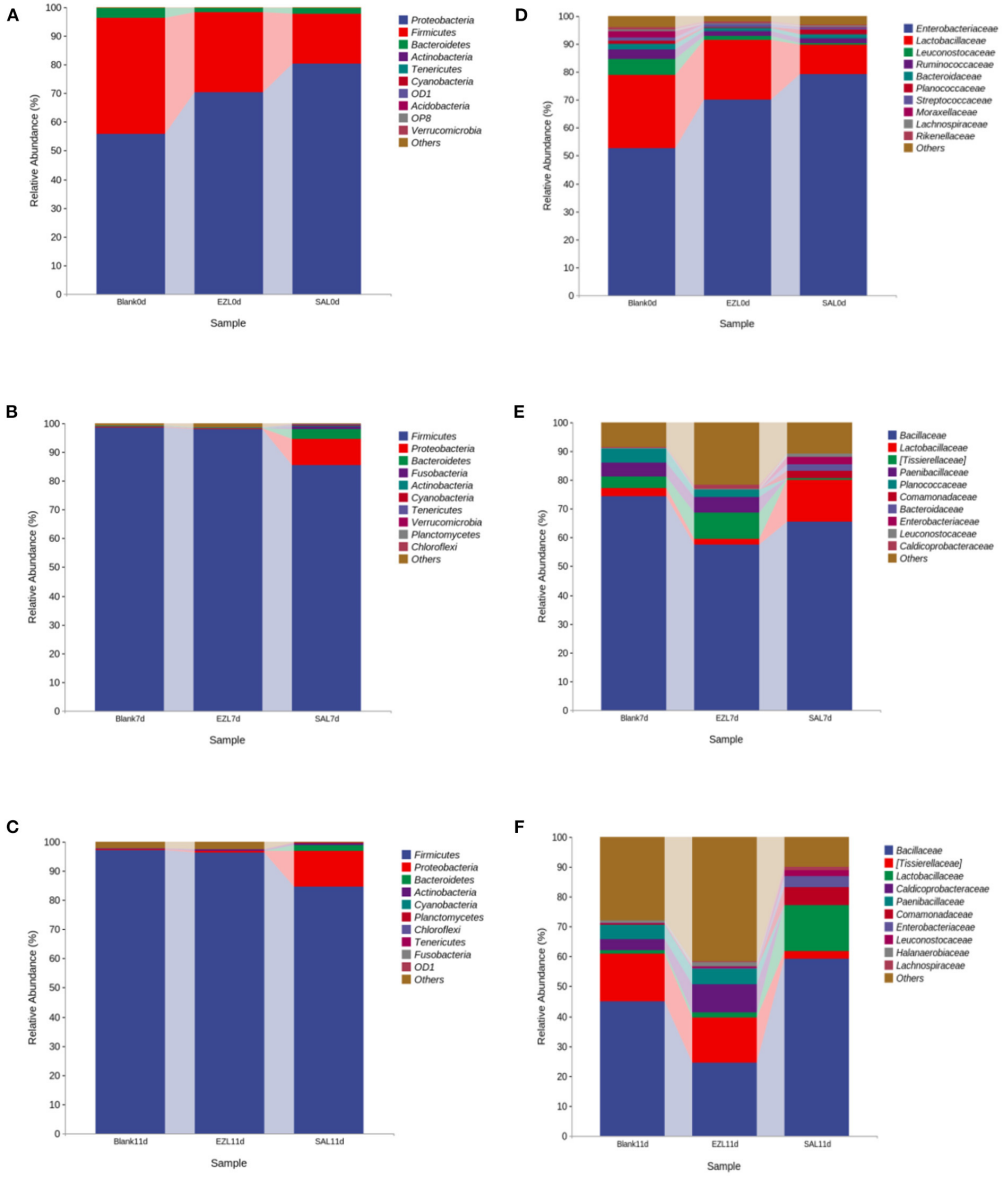
The dynamic relative abundance of the top 10 bacteria in taxonomic distribution at the phylum and the family levels during manure composting. **(A–C)** were the abundances in the phylum level; **(D–F)** were the abundances in the family level; **(A,D)** were the day 0; **(B,D)** were the day 7; **(C,F)** were the day 11, respectively. Blank was the control group; SAL was the salinomycin group; ZEL was the ethanamizuril group.

To elucidate the effect of two coccidiostats on the composition of the manure microbiota, we analyzed the bacteria at the phylum and family levels to characterize the dynamics of microbial taxonomic distribution (Figure 5). At the phylum level, *Proteobacteria* (55.8%), *Firmicutes* (40.4%), and *Bacteroidetes* (3.3%) dominated the manure microbial community in all three groups on day 0. Compared to the control group, the relative abundance of *Proteobacteria* (80.4%) was significantly increased (*p* < 0.05) and *Firmicutes* (17.4%) was significantly decreased in the salinomycin group (*p* < 0.05), whereas *Bacteroidetes* was significantly decreased both in the ethanamizuril group (1.6%) and in the salinomycin group (1.9%) (*p* < 0.05) (Figure 5A). On days 7 and 11, *Firmicutes* were the absolutely dominant bacteria in all groups (98.7% in the control group, 98% in the ethanamizuril group, and 85.4% in the salinomycin group). The relative abundance of *Proteobacteria* in the salinomycin groups was 9.1% on day 7 and 12.2% on day 11, where both were significantly higher than those of the other groups (*p* < 0.05) (Figures 5B,C). At the family level, the bacterial composition was further compared in the manure. *Enterobacteriaceae, Lactobacillaceae, Leuconostocaceae, Ruminococcaceae*, and *Bacteroidaceae* dominated the manure microbial community on day 0. Compared to the control group, the relative abundance of *Enterobacteriaceae* was significantly increased (*p* < 0.05), whereas *Lactobacillaceae, Leuconostocaceae*, and *Ruminococcaceae* were significantly decreased (*p* < 0.05) in the ethanamizuril group and the salinomycin group (Figure 5D). On days 7 and 11, *Bacillaceae* was the absolutely dominant bacteria in all groups, and there was no significant difference in microbial abundance among the groups.

### Impact on the loam microbiota

**Figure 6:**
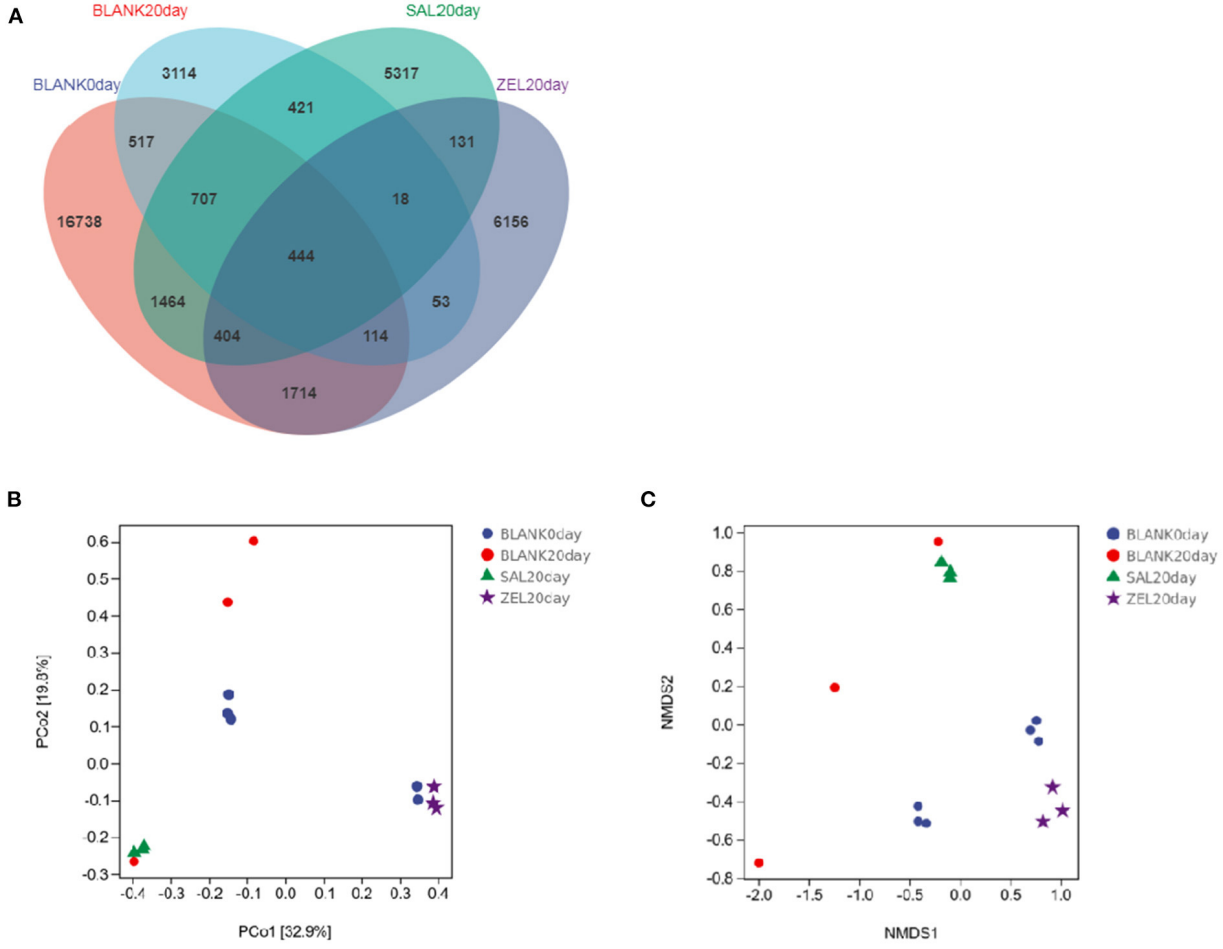
Venn diagram of shared OTUs of the different groups, and Principal coordinates analysis (PCoA) and Non-Metric Multi-Dimensional Scaling (NMDS) of the structure of the loam soil microbiota. **(A)** for Venn diagram; **(B)** for PCoA; **(C)** for NMDS, respectively. Blank 0 day was the control group on day 0; Balnk 20 day was the control group on day 20; SAL 20 day was the salinomycin group; ZEL 20 day was the ethanamizuril group.

The effect of salinomycin and ethanamizuril on the microbiota profiles of loam soil was analyzed by 16S ribosomal amplicon sequencing. Following bacterial DNA isolation and sequencing of the V3–V4 region of the 16S rRNA gene, a total of 7,767,847 high-quality reads were obtained. The average assembled 16S (V3–V4) length was 420 nucleotides, ranging from 208 to 442 bp. In total, 31,769 OTUs were observed in the four experimental groups, of which 444 OTUs were common to every group (Figure 6A). Both the numbers of total and unique OTUs in the control group on day 0 (BLANK 0 day) were the most, while those in the control group on day 20 group (BLANK 20 day) were the least.

As presented in Table 3, compared with the control group on day 0 (BLANK 0day), all alpha diversity indicators related to richness, diversity, and evenness (BLANK 20 day) were decreased significantly after 20 days (*p* < 0.05). The Shannon index and the Simpson index of the loam microbiota treated with salinomycin (SAL 20 day) were decreased significantly too (*p* < 0.05), but there was no significant difference in the control group on day 20 group (BLANK 20 day). On the other hand, the Shannon index and Simpson index of the loam microbiota treated with ethanamizuril (ZEL 20 day) were significantly higher than those of the control group on day 20 (BLANK 20 day), but there was no significant difference from the control group on day 0 group (BLANK 0 day). To further reveal the differences in soil microbiota composition among individual treatments, beta diversity indicators were determined using PCoA and NMDS plot. As shown in Figures 6B,C, the PCoA and NMDS score plots revealed that there was remarkable segregation in the soil microorganism between the control group on day 0 (BLANK 0day) vs. the control group on day 20 group (BLANK 20 day) and the salinomycin group (SAL 20 day). Similarly, the plots of the control group on day 20 group (BLANK 20 day) were significantly separated from those of the ethanamizuril group (ZEL 20 day). The results of beta diversity confirmed the earlier observation on alpha diversity.

**Table 3:** Alpha diversity indexes of the loam soil microbiota.

**Group**	**Chao1**	**Faith_pd**	**Goods_coverage**	**Observed_species**	**Pielou_e**	**Shannon**	**Simpson**
BLANK0day	5,174.8 ± 1,264.5^a^	331.65 ± 57.39^a^	0.99 ± 0.00	4,959.7 ± 1,132.6^a^	0.85 ± 0.06^a^	10.37 ± 0.94^a^	0.99 ± 0.01^a^
BLANK20day	2,142.6 ± 1,418.6^b^	189.15 ± 80.75^b^	0.99 ± 0.01	1,987.7 ± 1,272.7^b^	0.61 ± 0.08^b^	6.48 ± 0.54^b^	0.94 ± 0.03^b^
SAL20day	4,005.8 ± 427.2^ab^	279.59 ± 18.41^ab^	0.99 ± 0.00	3,674.4 ± 373.6^ab^	0.66 ± 0.03^b^	7.83 ± 0.50^b^	0.96 ± 0.01^b^
ZEL20day	3,979.4 ± 153.1^ab^	254.12 ± 11.22^ab^	0.99 ± 0.00	3,860.9 ± 174.8^a^	0.83 ± 0.01^a^	9.88 ± 0.11^a^	0.99 ± 0.00^a^

BLANK0day, control group on day 0; BLANK20day, control group on day 20; ZEL20day, ethanamizuril group; SAL20day, salinomycin group. Differentt letters in the same column showed statistically significant difference in the same index (p < 0.05).

**Figure 7:**
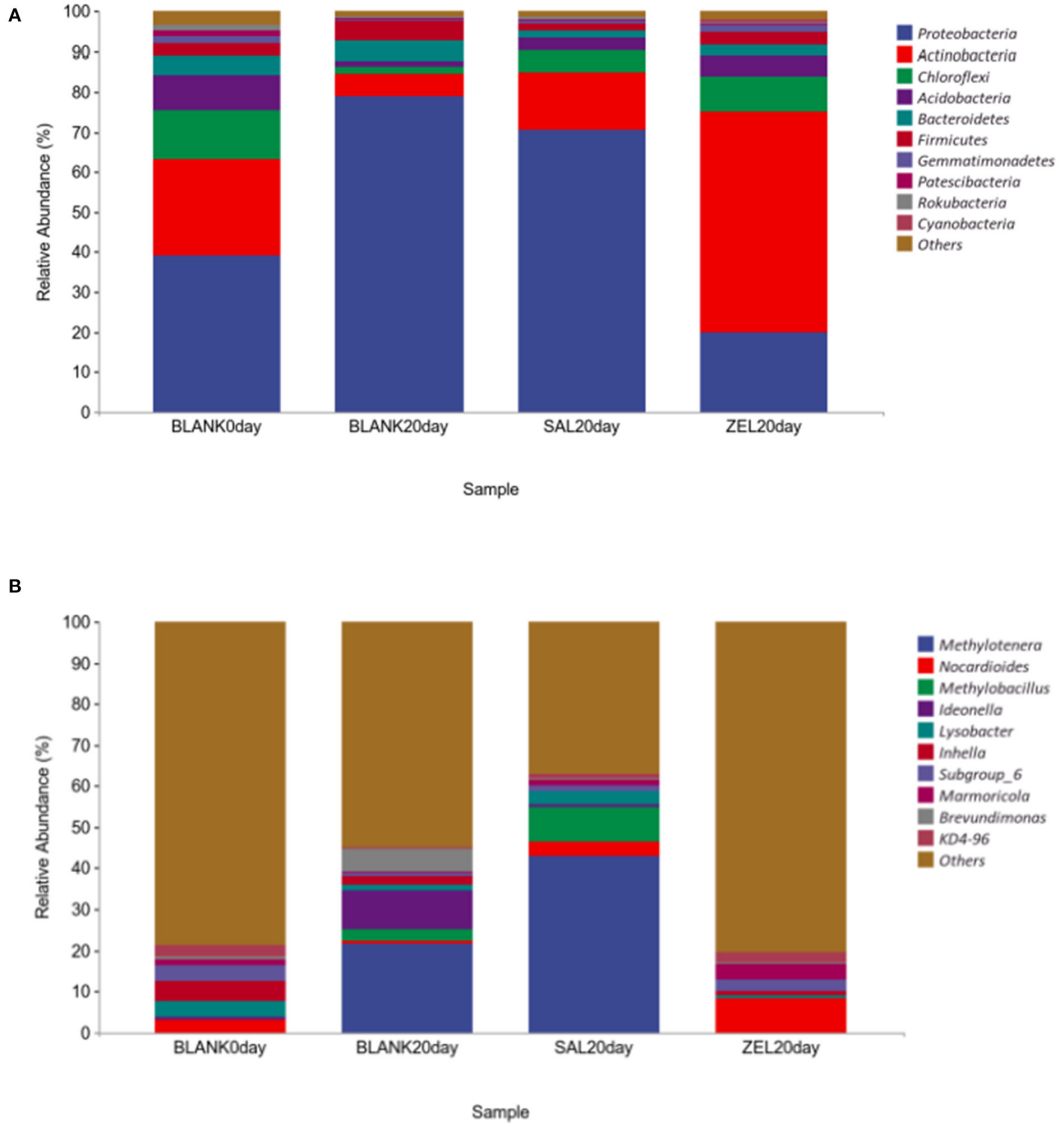
The relative abundance of the top 10 bacteria in microbial taxonomic distribution at the phylum and the genus levels in loam soil. **(A)** was the abundance in the phylum level; **(B)** was the abundance in the genus level. The same color in each diagram represented the same bacteria, and different areas represented different proportions. Blank 0 day was the control group on day 0; Balnk 20 day was the control group on day 20; SAL 20 day was the salinomycin group; ZEL 20 day was the ethanamizuril group, respectively.

To elucidate the effect of two coccidiostats on the composition of the loam soil microbiota, we analyzed the bacteria at the phylum and genus levels to characterize the dynamics of microbial taxonomic distribution (Figure 7). At the phylum level (Figure 7A), *Proteobacteria, Actinobacteria, Chloroflexi, Acidobacteria*, and *Bacteroidetes* dominated the soil microbial community in the control group on day 0 (BLANK 0 day), and the relative abundance of *Proteobacteria, Actinobacteria, Chloroflexi*, and *Acidobacteria* were 39, 24, 12, and 9%, respectively. The relative abundance of *Proteobacteria* in the control group on day 20 (BLANK 20 day) and the salinomycin group (SAL 20 day) sharply increased to 79% (*p* < 0.05) and 71% (*p* < 0.05) and decreased to 20% (*p* < 0.05) in the ethanamizuril group (ZEL 20 day). The relative abundance of *Actinobacteria* in the control group on day 20 (BLANK 20 day) and the salinomycin group (SAL 20 day) sharply decreased to 5 (*p* < 0.05) and 15% (*p* < 0.05) and increased to 55% (*p* < 0.05) in the ethanamizuril group (ZEL 20 day). The relative abundance of *Chloroflexi* and *Acidobacteria* all decreased in the other three groups. At the genus level (Figure 7B), there was no bacteria genus of which relative abundance exceeded 5% in the control group on day 0 (BLANK 0 day). Similarly, only *Nocardioides* exceeded 5% in the ethanamizuril group (ZEL 20 day). However, the proportion of *Methylotenera* reached 22 (*p* < 0.05) and 43% (*p* < 0.05) in the control group on day 20 (BLANK 20 day) and the salinomycin group (SAL 20 day) respectively, and *Methylobacillus* were increased significantly too (*p* < 0.05).

**Figure 8:**
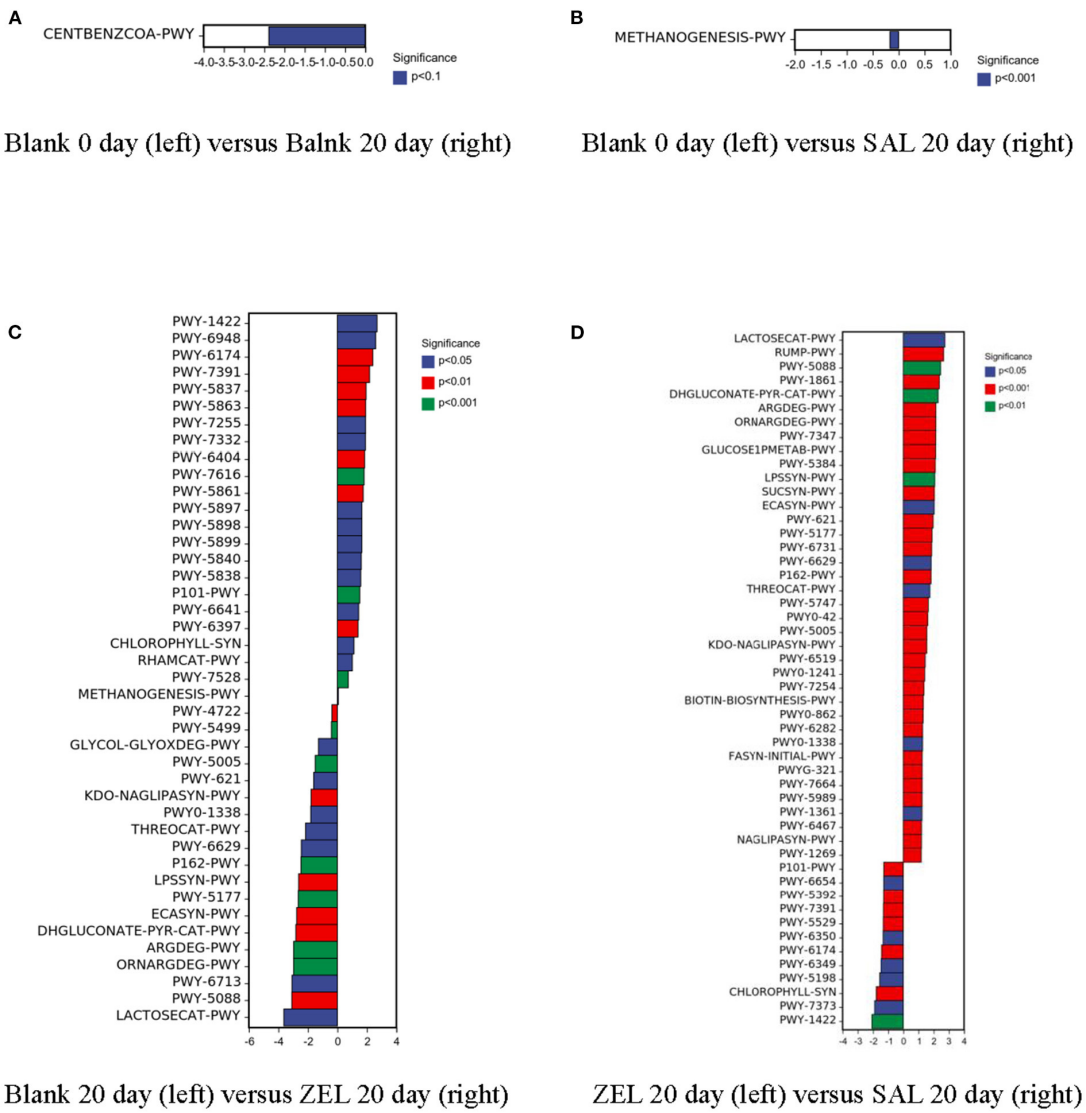
Predicted analysis for metabolic functional abundance of significant differences in samples based on sequence abundance in loam soil. Blank 0 day was the control group on day 0; Balnk 20 day was the control group on day 20; SAL 20 day was the salinomycin group; ZEL 20 day was the ethanamizuril group. **(A)** for Balnk 20 day (right) vs. Blank 0 day (left); **(B)** for Balnk 20 day vs. SAL 20 day; **(C)** for ZEL 20 day vs. Balnk 20 day; **(D)** for SAL 20 day vs. ZEL 20 day, respectively.

Furthermore, we tried to use sequence abundance to predict the metabolic functional abundance of significant differences in samples. According to Figure 8, compared with the control group on day 0 (BLANK 0 day), benzoyl-CoA degradation II (anaerobic) (CENTBENZCOA-PWY) was upregulated significantly in the control group on day 20 (BLANK 20day). Compared with the control group on day 20 (BLANK 20 day), methanogenesis from H_2_ and CO_2_ (METHANOGENESIS-PWY) was upregulated significantly in the salinomycin group (SAL 20 day). When the ethanamizuril group was compared with the control group on day 20 (BLANK 20 day), there were 23 significant upregulation and 19 significant downregulation pathways, including creatinine degradation II, methanogenesis from H_2_ and CO_2_, lactose and galactose degradation I, and so on. In the ethanamizuril group vs. the salinomycin group, there were 50 significantly different metabolic pathways too.

### Impact on physiological profiling of soil microbial community

**Figure 9:**
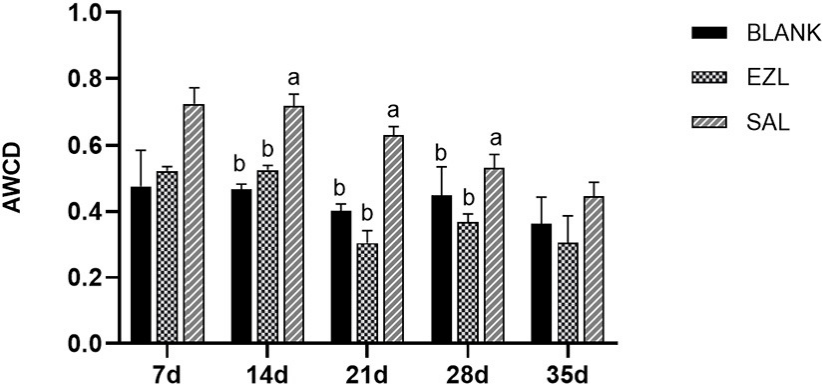
Average well-color development (AWCD) of BIOLOG ECO microplates within incubation time of microbial communities from loam of control group (BLANK), ethanamizuril group (EZL) and salinomycin group (SAL) for days 7, 14, 21, 28 and 35. Different letters on the column showed statistically significant differences on the same day (*p* < 0.05).

To evaluate the metabolic activity of loam microbial communities exposed to salinomycin and ethanamizuril, the development of AWCD of all carbon sources was investigated. All samples showed an increase in AWCD at all stages, indicating that microbial communities from the loam samples could metabolize carbon substrates in BIOLOG ECO microplates. As shown in Figure 9, the AWCD value of the salinomycin group was the highest at all five time points and significantly higher than that of the control group and the ethanamizuril group on days 14, 21, and 28 (*p* < 0.05). There was no significant difference between the ethanamizuril group and the control group concerning AWCD at all stages (*p* > 0.05). Overall, the AWCD values of each group showed a downward trend with the extension of the incubation time of the loam sample. Furthermore, the utilization of L-serine, L-asparagine, γ-hydroxybutyric acid, N-acetyl-D-glucamine, 4-hydroxybenzoic acid, and D-galactose in the salinomycin group, and L-asparagine and putrescine in the ethanamizuril group were significantly greater than those in the control group on some days (*p* < 0.05). However, the utilization of the L-arginine, γ-hydroxybutyric acid, D-galacturonic acid, D-glucuronic acid, and N-acetyl-D-glucamine in the ethanamizuril group was significantly lower than those in the control group on some days (*p* < 0.05).

**Figure 10:**
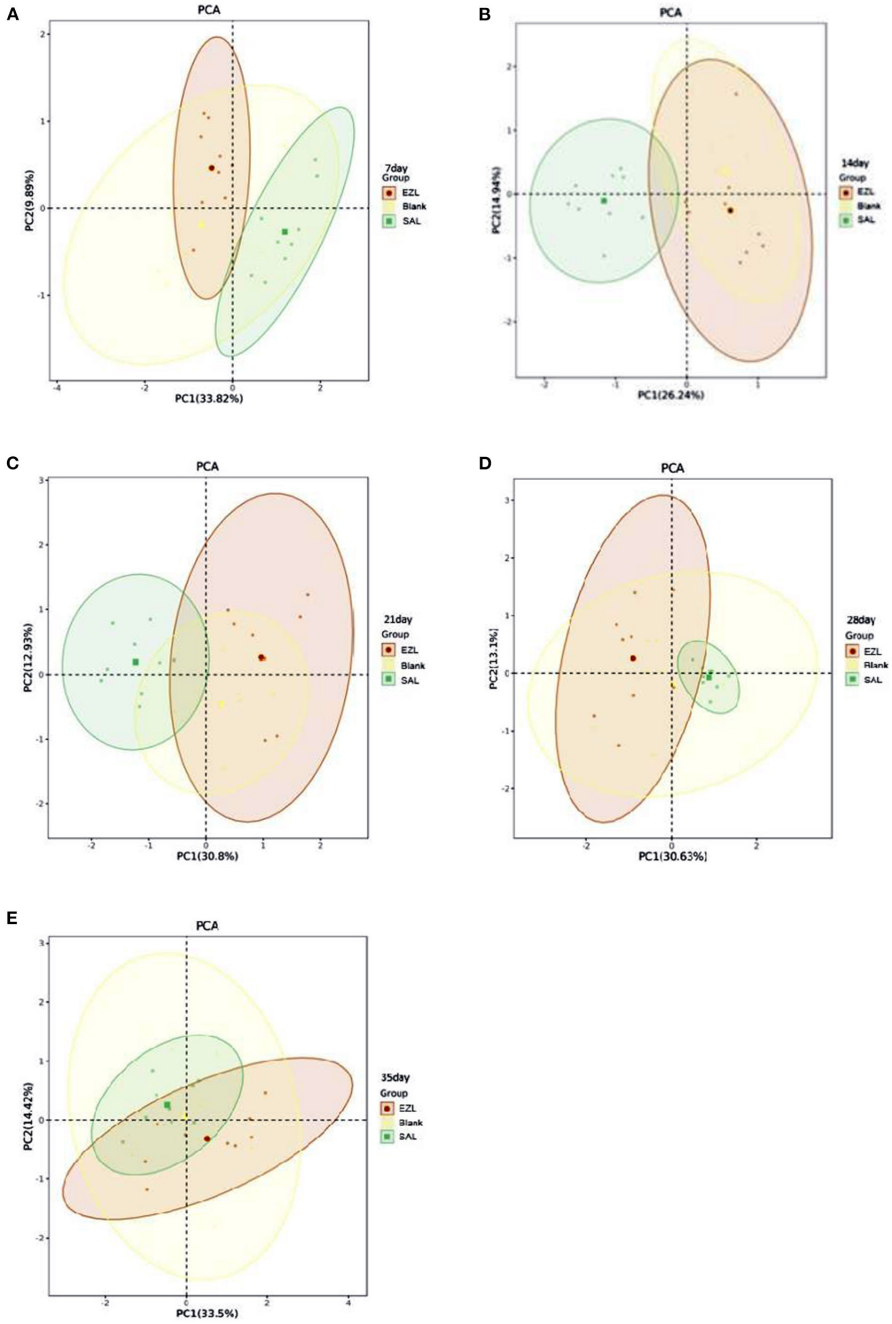
The plot of principal component analysis for the soil microbial carbon source utilization. Blank: control group; EZL: ethanamizuril group; SAL: salinomycin group. **(A–E)** were incubation time for 7, 14, 21, 28 and 35 days, respectively.

The metabolic functional diversity after different treatments was evaluated by the Shannon, Simpson, and McIntosh indices. As presented in Table 4, the three indices of the salinomycin group were the highest at each sampling time among the three groups. Except on day 35, both the Shannon and Simpson indices of the salinomycin group were significantly higher than the control group (*p* < 0.05). There was no significant difference between the ethanamizuril group and the control group. Moreover, PCA revealed significant different positions among groups on carbon utilization characteristics. Figure 10 indicates that the point distributions of the ethanamizuril group and the control group in the PCA plot were relatively scattered and overlapped with each other. The point position of the salinomycin group was obviously clustered in one sector and distinct from the other groups on days 14 and 21.

**Table 4:** Metabolic functional diversity index of microbial community of soil.

**Index**	**Group**	**Sampling time (day)**				
		**7**	**14**	**21**	**28**	**35**
Shannon	BLANK	2.79 ± 0.20^b^	2.75 ± 0.03^b^	2.58 ± 0.08^b^	2.68 ± 0.36^ab^	2.55 ± 0.38
	EZL	2.86 ± 0.06^b^	2.74 ± 0.04^b^	2.38 ± 0.14^b^	2.40 ± 0.10^b^	2.47 ± 0.31
	SAL	3.30 ± 0.18^a^	3.16 ± 0.04^a^	3.05 ± 0.06^a^	2.96 ± 0.07^a^	2.94 ± 0.05
Simpson	BLANK	0.93 ± 0.02^b^	0.93 ± 0.00^b^	0.91 ± 0.01^ab^	0.92 ± 0.03^ab^	0.90 ± 0.04
	EZL	0.93 ± 0.00^ab^	0.93 ± 0.00^b^	0.89 ± 0.02^b^	0.89 ± 0.02^b^	0.89 ± 0.03
	SAL	0.95 ± 0.00^a^	0.95 ± 0.00^a^	0.95 ± 0.00^a^	0.94 ± 0.00^a^	0.94 ± 0.00
Mclntosh	BLANK	3.90 ± 0.96	3.91 ± 0.22^b^	3.62 ± 0.15^b^	3.79 ± 0.49	3.27 ± 0.83
	EZL	4.13 ± 0.29	4.38 ± 0.22^ab^	3.07 ± 0.50^b^	3.75 ± 0.24	2.93 ± 0.91
	SAL	4.94 ± 0.79	4.83 ± 0.40^a^	4.52 ± 0.32^a^	3.99 ± 0.40	3.42 ± 0.58

Blank, control group; EZL, ethanamizuril group; SAL, salinomycin group. Differentt letters in the same column showed statistically significant difference in the same index (P < 0.05).

## Discussion

This study was the first to investigate and compare the effects of salinomycin and ethanamizuril on the microbiota of coccidia-infected broilers' cecal content, manure composting, and loam soil. The current research results figure out that salinomycin and ethanamizuril have different effects on the composition of different microbial flora. Furthermore, the change in microbial composition could affect the micro-ecological function.

*Firmicutes, Actinobacteria, Bacteroidetes*, and *Proteobacteria* are the main phyla in poultry intestines (Waite and Taylor, 2015). Coccidia is the major intestinal protozoa of birds, which directly or indirectly affects the health of the host and significantly decreases microbiota diversity in the host intestine, and changes in the intestinal microbiota may also influence the infectivity of coccidian (Lu et al., 2021). The intestinal microbiome composition can be influenced by drugs too. It has been also reported that salinomycin and other ionophores could lead to a significant increase in evenness and a drastic decrease in the richness of the cecal content microbiota in broilers (Trela et al., 2020). Infection with *E. tenella* would lead to a decrease in the abundance of most bacterial taxa in chicken intestines except for members of the family Enterobacteriaceae (Kimura et al., 1976). In this study, the Shannon and Simpson indices of the cecum contents were significantly decreased, and the abundance of *Escherichia-Shigella* was obviously increased, whereas *Faecalibacterium* of *Firmicutes* and *Bacteroidota* of *Alistipes* decreased in response to the *E. tenella* infection. These data indicate that the *Eimeria* infection might strongly cause dysbiosis or disruption of resident microbiota and contribute to subsequent infection by other pathogens. It did not reverse the disturbance of cecal content microbiota, although the treatments of ethanamizuril or salinomycin significantly reduced chicken cecal lesions and oocyst shedding with showing high anticoccidial activity (data were not shown). Strikingly, some bacteria of *Firmicutes* such as *UCG-005, Enterococcus*, and *Streptococcus* tended to be consistent with that of the healthy group after ethanamizuril and salinomycin treatment. Compared to the non-medicated control group, *Lampropedia, Paracoccus, Neomegalonema, Gemmobacter*, and *Brevundimonas* were significantly decreased both in the salinomycin group and the ethanamizuril group. Furthermore, *Enterobacter* and *Acinetobacter* that have been reported as opportunistic pathogens in plants, animals, and humans and contain the major resistant bacterial pathogens (Davin-Regli et al., 2019; Shin et al., 2020), were significantly decreased in the salinomycin group. The similarity of the contribution of bacterial taxa to the difference of bacterial clustering suggested that the treatment of salinomycin and ethanamizuril may have similar effects on coccidia-infected cecal microbiota. However, salinomycin had better inhibitory effect on opportunistic pathogenic bacteria because of its extensive and strong antibacterial activity. It is generally accepted that the microbiota associated with health must have a degree of resilience to external or internal disturbances. Given that coccidiostats treatment showed the high therapeutic effects on coccidian infection and decreased some pathogenic bacteria in this study, we believe that salinomycin and ethanamizuril promoted the transformation of chicken cecal microbiota into a new equilibrium state that is facilitated by the different anticoccidial mechanisms of salinomycin and ethanamizuril.

Intestinal contents include gut flora metabolites and host metabolites, which are the key substances connecting the host metabolome and microbiome (Matsumoto et al., 2012). *Eimeria* infection could result in the alterations of key metabolites related to fatty acid metabolism, nucleotide metabolism, amino acid metabolism, and inflammatory reaction (Aggrey et al., 2019). Based on the microbiota results of 16S RNA sequencing, we predicted that the chemoheterotrophy and fermentation in the control group were significantly greater than those of the other three groups. This hypothesis had been confirmed in metabolomic research that showed the number of differential metabolites of the cecum contents in all chickens infected with *E. tenella* increased surprisingly when compared to the healthy group. Treatments of both salinomycin and ethanamizuril failed to improve the trend of the number increasing in differential metabolites, which indicated that *Eimeria* infection has a great impact on cecum flora metabolism and host metabolism. Furthermore, the metabolites of chicken infected with coccidia changed by ethanamizuril were also different from that of salinomycin. Ethanamizuril mainly promoted o-cresol and other 48 metabolites increased, and L-methionine and other 25 metabolites decreased. These differential metabolites were mainly involved in purine metabolism, aminoacyl-tRNA biosynthesis, and other 6 metabolic pathways, which suggested that ethanamizuril may play an anticoccidian role by inhibiting the biosynthesis of cellular protein components. Moreover, metabolites such as peptidomimetics, amines, guanidine, fatty acyls interfered with salinomycin, and histidine metabolism and the other 9 metabolic pathways were perturbed. The involvement of the histidine metabolism pathway suggested that salinomycin may act against coccidia invasion by promoting intestinal innate immune response (Yamaki et al., 1998).

Over the past decades, composting has been increasingly recognized as one of the “best available techniques” for the on-farm processing of manure (Esperon et al., 2020). It is estimated that ~75% of the drugs are not absorbed by animals and are excreted with feces, which has prompted an interest on their fate and their impact on manure and soil microbiota communities (Chee-Sanford et al., 2009). *Firmicutes, Proteobacteria, Bacteroidetes*, and *Actinobacteria* are the four dominated bacterial phyla in initial composting microbiota, which represent at least three-quarters of the total bacterial community. The microbiota state of initial composting could have persistent effects for later phases of composting. Nevertheless, the compositions of the bacterial community were mainly influenced by redox potential, pH, and moisture, whereas temperature was another main environmental factor (Mao et al., 2020). Drastic changes in community composition and a clear reduction in bacterial and fungal diversity were observed during composting, which were strongly linked with both physicochemical compost properties and microbial community composition. Using classical microbiology methods, some key bacteria (*Salmonella spp*., *Campylobacter spp*., and *Escherichia coli*) were found to be reduced, which may be attributed to the high temperatures generated during composting that are lethal for most pathogenic bacteria (Erickson et al., 2015). In this study, *Firmicutes, Proteobacteria*, and *Bacteroidetes* dominated in manure microbiota during the initial composting, whereas the relative abundance of *Proteobacteria* was substantially increased and *Bacteroidota* was decreased compared with the chicken cecal bacterial composition of the control group. This phenomenon may be attributed to the contamination and rapid reproduction of *Proteobacteria* caused by the excreted feces being exposed to the breeding environment. *Firmicutes* gradually gained absolute dominance in the community composition of each treatment group following composting progress. This could be explained by conditions (pH, temperature program, moisture, and electrical conductivity) of this study were more suitable for the reproduction of *Firmicutes* phylum, and the ability to form endospores that can help it to survive high temperatures and harsh environments (Bello et al., 2020). A rise in pH and electrical conductivity indicated that the microbiota decompose the organic substance of manure and release various small molecules during the composting process. Strikingly, *Proteobacteria* always occupied a certain dominant proportion in the salinomycin group during the composting process. *Proteobacteria* phylum is formed by Gram-negative bacteria and includes a wide variety of pathogenic species such as *Escherichia spp., Campylobacter spp., Salmonella spp., and Pseudomonas spp*. A recent study has shown that some members of *Proteobacteria* can act as a reservoir for the dissemination of resistance to antibiotics in other pathogenic bacteria (Macdonald et al., 2017). The high percentage of *Proteobacteria* phylum in the salinomycin group may indicate that salinomycin is detrimental to the composting environment and may induce the risk of pathogen transmission. Furthermore, *Bacteroidetes* and microbiota richness were significantly decreased in both salinomycin and ethanamizuril groups at the initial composting. *Bacteroidetes* phyla are considered the primary degraders of polysaccharides (Lapebie et al., 2019). These data suggested that the decomposition of polysaccharide substances in the feces excreted by the two coccidiostats-treated chickens may be briefly inhibited during manure composting, but the influence on long-term composting is limited.

Soil microorganisms are the main drivers of organic substance transformation in soil, which have a significant impact on the level of soil fertility. The disparity in soil properties has a considerable impact on soil microbiome constituents. It has been reported that the introduction of pesticides into the soil environment can initiate processes that promote or inhibit soil microbial activity and alter the structure of the microbial community (Medo et al., 2021). The evenness and richness of the microbiota in the control group and the salinomycin group decreased significantly under natural conditions, indicating that the composition of the soil microbial community is a dynamic process and is affected by multiple factors. *Proteobacteria* phylum possesses a robust capacity to respond to carbon deficiencies and is reckoned as an abundant bacterial group in soil (Muller et al., 2016). *Proteobacteria* sharply increased in the control group on day 20, and the salinomycin group indicated that the carbon source in the soil may be relatively deficient, and other bacterial phyla have difficulty in growing with limited carbon. It was confirmed by the upregulation of benzoyl-CoA degradation II and methanogenesis from H_2_ and CO_2_ in functional predictions based on the sequence abundance. Interestingly, combined with the inhibition of *Proteobacteria* by salinomycin in the manure composting, we speculate that salinomycin may have a mechanism to promote its reproduction. Referring to the development of the control group, the progression of taxonomic abundance was obviously delayed by the ethanamizuril treatment. The *Actinobacteria* phylum is one of the oldest bacterial phyla and is a well-recognized biosynthetic factory that produces abundant antibiotics (Jose et al., 2021). *Actinobacteria* increased significantly with the ethanamizuril treatment, suggesting that ethanamizuril may promote the growth of *Actinobacteria* and may produce certain secondary metabolites, which compete and inhibit the reproduction of other taxonomic bacteria.

The 16S ribosomal amplicon sequencing focuses on characterizing the species, number, structure, and diversity of soil microbial communities, but is limited in terms of physiological profiling. The BIOLOG ECO microplate is a relatively simple and quick method for characterizing the ecological diversity and community-level status of microbial populations, which is based on metabolic functions using a redox system with 31 different sole carbon sources (Gryta et al., 2014). It has been reported that the BIOLOG ECO microplate was used to reveal the ecological status of environmental samples, such as soils, activated sludge, and sediments (Al-Dhabaan and Bakhali, 2017). The versatility derived from the BIOLOG ECO microplate has the potential to distinguish the effects of salinomycin and ethanamizuril on the metabolic activity of loam microbial communities. In this study, the AWCD decreased with the extension of incubation time, implying that the carbon and nitrogen sources in the loam were gradually depleted and the metabolic activity of loam microbiota declined gradually. However, AWCD of the salinomycin group exhibited obviously increased, which may be attributed to the improvement of metabolic capacity by salinomycin treatment. This result was consistent with the significant increase of *Proteobacteria* phylum in the salinomycin group observed in rDNA amplicon analysis. Based on the clustering of different carbon source utilization, alpha diversity analysis showed that the evenness and the richness were significantly increased in the salinomycin group, and the PCA calculation showed that the carbon source utilization mode of salinomycin treatment was different from that of the control group and the ethanamizuril group at an early stage. These data indicated that salinomycin may interfere with the metabolic function of soil microbiota, which should be attributed to its disruptive effects on soil microbiota composition in rDNA amplicon analysis. It is noteworthy that there was almost no significant difference between the ethanamizuril group and the control group in all assessment indicators, such as AWCD and alpha diversity, indicating that ethanamizuril has a very mild effect on the metabolism of soil microbiota.

Previous studies have confirmed that salinomycin exhibits a broad-spectrum antibiotic activity by destroying cell membrane ion transport (Miyazaki et al., 1974), whereas ethanamizuril has only anticoccidial activities but no antibacterial potential (data were not shown). The differences in antibacterial activity contribute to revealing that the interactions between salinomycin and various microbial communities were more violent than those of ethanamizuril. In addition, the degradation rate of salinomycin is relatively rapid in manure and soil (Sun et al., 2014), whereas ethanamizuril is insoluble in water and is stable in soil and manure (data were not shown). Therefore, these potential biological and physicochemical characteristics may support this perspective that the interaction between salinomycin and bacterial community led to the decrease of salinomycin concentration, and the influence on microbiota was limited to the early stage.

Our study reveals that salinomycin and ethanamizuril have different action patterns on various microbial communities for the first time. In the animal model of coccidia infection, the pathogen is the main driving force that affects the microbiota and metabolites of the chicken cecum, whereas the treatment of salinomycin and ethanamizuril may reconstruct a new equilibrium of the intestinal microbiota. In an *in vitro* environment, the effect of ethanamizuril on composting and soil microbiota seems to be slight. However, salinomycin has a great impact on the microbial communities of manure composting and soil, which promote the utilization of carbon sources. In particular, the promoting effect of salinomycin on the *Proteobacteria* phylum should be further researched. In conclusion, all the data of this study will contribute to elucidating the interaction between anticoccidial drugs and microbial communities and contribute to the using and dealing of salinomycin and ethanamizuril in the poultry industry scientifically.

## Data availability statement

The datasets presented in this study can be found in online repositories. The names of the repository/repositories and accession number(s) can be found at: NCBI - PRJNA865872, PRJNA848967.

## Ethics statement

The animal study was reviewed and approved by Shanghai Veterinary Research Institutional Animal Care Committee and in accordance with the National Institutes of Health Guide on the Care and Use of Laboratory Animals.

## Author contributions

KZ: conceptualization and funding acquisition. XW and WZ: project administration and resources. HZ, KZ, and XC: roles/writing the original draft, reviewing, and editing. CW and CF: validation. XC and CF: investigation and methodology. KZ and HZ: data curation and formal analysis. All authors contributed to the article and approved the submitted version.

## Funding

The work was supported by the National Key R&D Program of China (2018YFE0192600, 2018YFD0500302).

## Conflict of interest

The authors declare that the research was conducted in the absence of any commercial or financial relationships that could be construed as a potential conflict of interest.

## Publisher's note

All claims expressed in this article are solely those of the authors and do not necessarily represent those of their affiliated organizations, or those of the publisher, the editors and the reviewers. Any product that may be evaluated in this article, or claim that may be made by its manufacturer, is not guaranteed or endorsed by the publisher.
