# Supplementary material for: Effects of salinomycin and ethanamizuril on the three microbial communities in vivo and in vitro
Source: Front Microbiol. 2022 Aug 12;13:941259. doi: 10.3389/fmicb.2022.941259 (PMC9413843; doi:10.3389/fmicb.2022.941259)
Supplement: Supplementary file 1 [file Table_1.docx]

**Table S**

**Table S1 Basic physical and chemical properties of manure samples during composting**

| **Group** | **Time (day)** | **pH** | **Electrical conductivity (µs/cm)** | **Water content (%)** |
| --- | --- | --- | --- | --- |
|  | 0 | 6.1 | 2685.9 | 71.94 |
| Blank | 7 | 6.9 | 4077.2 | 72.90 |
|  | 11 | 9.0 | 4233.8 | 78.70 |
|  | 0 | 6.0 | 2605.2 | 70.62 |
| EZL | 7 | 8.6 | 3835.4 | 76.59 |
|  | 11 | 7.2 | 4081.4 | 80.80 |
|  | 0 | 6.4 | 2618.4 | 74.38 |
| SAL | 7 | 5.9 | 4494.2 | 74.46 |
|  | 11 | 9.1 | 4125.4 | 76.20 |

Note: Blank: control group; EZL: ethanamizuril group; SAL: salinomycin group.
